# Impacts of CA9 Gene Polymorphisms on Urothelial Cell Carcinoma Susceptibility and Clinicopathologic Characteristics in Taiwan

**DOI:** 10.1371/journal.pone.0082804

**Published:** 2013-12-13

**Authors:** Shian-Shiang Wang, Yu-Fan Liu, Yen-Chuan Ou, Chuan-Shu Chen, Jian-Ri Li, Shun-Fa Yang

**Affiliations:** 1 Institute of Medicine, Chung Shan Medical University, Taichung, Taiwan; 2 Division of Urology, Department of Surgery, Taichung Veterans General Hospital, Taichung, Taiwan; 3 Department of Biomedical Sciences, Chung Shan Medical University, Taichung, Taiwan; 4 Department of Medical Research, Chung Shan Medical University Hospital, Taichung, Taiwan; China Medical University, Taiwan

## Abstract

**Background:**

Carbonic anhydrase 9 (CA9) is reportedly overexpressed in several types of carcinomas and is generally considered a marker of malignancy. The current study explored the effect of *CA9* gene polymorphisms on the susceptibility of developing urothelial cell carcinoma (UCC) and the clinicopathological status.

**Methodology and Principal Findings:**

A total of 442 participants, including 221 healthy people and 221 patients with UCC, were recruited for this study. Four single-nucleotide polymorphisms (SNPs) of the *CA9* gene were assessed by a real-time PCR with the TaqMan assay. After adjusting for other co-variants, the individuals carrying at least one A allele at *CA9* rs1048638 had a 2.303-fold risk of developing UCC than did wild-type (CC) carriers. Furthermore, UCC patients who carried at least one A allele at rs1048638 had a higher invasive stage risk (p< 0.05) than did patients carrying the wild-type allele. Moreover, among the UCC patients with smoker, people with at least one A allele of *CA9* polymorphisms (rs1048638) had a 4.75-fold (95% CI = 1.204–18.746) increased risk of invasive cancer.

**Conclusion:**

The rs1048638 polymorphic genotypes of *CA9* might contribute to the prediction of susceptibility to and pathological development of UCC. This is the first study to provide insight into risk factors associated with *CA9* variants in carcinogenesis of UCC in Taiwan.

## Introduction

Urothelium covers the epithelial lining of the urinary tract from the renal calyces to the bladder. Any neoplastic lesion grew from the urothelium has similar behavior[1]. The most frequent malignant tumor of urinary tract is urothelial cell carcinoma (UCC). In the United State, bladder cancer is the fourth most common cancer among men [2] and bladder cancer is the second most genitourinary cancer. In Taiwan, bladder cancer is the ninth leading malignancy among men and the sixteenth leading malignancy among women [3]. UCC composed more than 90% of bladder cancer in both genders. The incidence of upper urinary tract UCC is also increasing. The most known risk factors are tobacco use and aromatic amines exposure [4]–[6]. However, many articles emphasized the importance of genetic factors in the development of UCC [7–10]. Genetic variation affects the function of oncogene, tumor suppressor gene or metabolism of enzyme to induce cancer formation. Hypoxia is the common cause of tumor [11]. Decreased O2 concentration created a hostile metabolic microenvironment (e.g., existence of severe tissue acidosis) to activate a variety of biological responses including signal pathways of proliferation, angiogenesis and death [11]. Tumor cells can survive and even grow under hypoxic condition. Tumor hypoxia is associated with aggressive tumor growth, metastasis, and treatment failure after radiation therapy and chemotherapy [12]. Hypoxia might be an important therapeutic target because it involved many different metabolic pathways in cancer growth.

Carbonic anhydrase (CA), a family of zinc metalloenzymes, can efficiently catalyze the reversible processes of hydration-dehydration of CO_2_ and HCO3^−^. Carbonic anhydrase 9 (CA9) is located on chromosome 9p12–13, which comprises 11 exons and encodes for the 459-amino-acidprotein. CA9 helps to keep a normal pH in tumor cells in a hypoxic microenvironment and allow tumor cell proliferation [13]. CA9, which is not expressed in most benign tissues, is abundant in many cancers including renal cancer, bladder cancer, oral cancer, lung cancer and uterine cancer and has been thought to be an endogenous marker for tumor hypoxia [13–20]. Single nucleotide polymorphism (SNP) is a variation in the DNA sequence that occurs when a nucleotide (A, T, C, or G) is changed in at least 1% of a certain population [13]. Previous study showed that SNP in the exon region of CA9 are associated with overall survival for metastatic renal cell carcinoma [21] and gene-environment interactions of CA9 polymorphisms, smoking, and betel quid chewing might alter oral cancer susceptibility and metastasis [13]. One published report suggests a tripartite role of CA9 as a diagnostic, prognostic, and therapeutic molecular marker in bladder cancer [16]. However, no reports focused on the association between CA9 gene polymorphisms and UCC. The present study investigated relationships between SNPs (rs2071676, rs3829078, and 376del393) in the exon and 3’-UTR (rs1048638) regions of the CA9 gene and the risk of UCC. The influences of these SNPs combined with tobacco consumption on clinicopathological characteristics of UCC were also evaluated. To our knowledge, this is the first study to demonstrate a significant association between CA9 gene polymorphisms and UCC.

## Materials and Methods

### Subjects and Specimen Collection

We recruited 221 patients (139 men and 82 women, with a mean age of 68.52 years) at Taichung Veterans General Hospital in Taichung, Taiwan. Patients were enrolled as a case group in 2011–2012. All patients have pathology proved urothelial cell carcinoma of upper urinary tract or bladder. Meanwhile, during the same study period, 221 age- and gender-matched individuals were enrolled as the controls that entered the physical examination at the same hospital. These control groups had neither self-reported history of cancer of any sites. Personal information and characteristics collected from the study subjects using interviewer-administered questionnaires contained questions involving demographic characteristics and the status of cigarette smoking. Before commencement of this study, approval was obtained from the Institutional Review Board (IRB) of Taichung Veterans General Hospital, and informed written consent to participate in the study was obtained from each person (IRB No. CF11094). For both cases and controls, we used a questionnaire to obtain information on patient exposure to tobacco consumption. Medical information for the cases was obtained from their medical records, and included TNM clinical staging, lymph node involvement, and histologic grade. UCC patients were clinically staged at the time of diagnosis according to the TNM staging system of the American Joint Committee on Cancer (AJCC) Staging Manual (7th ed.) Tumors are classified as superficial tumor (pT0-1, n = 138) and invasive tumor (pT2-4, n =  83). Metastasis into lymph nodes was detected in 11 cases (6.1%) and one patient has distal metastasis (0.6%). Whole-blood specimens collected from controls and UCC patients were placed in tubes containing ethylenediaminetetraacetic acid (EDTA), immediately centrifuged, and stored at –80°C [22].

### Genomic DNA extraction

Genomic DNA was extracted using QIAamp DNA blood mini kits (Qiagen, Valencia, CA, USA) following the manufacturer’s instructions. We dissolved DNA in TE buffer (10 mM Tris and 1 mM EDTA; pH 7.8) and then quantified it by measuring the OD260. The final preparation was stored at –20°C and was used to act as templates for the polymerase chain reaction (PCR) [23].

### Real-time PCR

Allelic discrimination of the *CA9* +201 (rs2071676), +1081 (rs3829078) and +1584 (rs1048638) allelic polymorphisms were assessed with an ABI StepOne™ Real-Time PCR System (Applied Biosystems, Foster City, CA, USA), and analyzed with SDS vers. 3.0 software (Applied Biosystems) using the TaqMan assay [24]. Furthermore, the 376del393 allelic polymorphisms were assessed with PCR. The products were separated on a 3% agarose gel and then stained with ethidium bromide [25]. The final volume for each reaction was 5 µL, containing 2.5 µL TaqMan Genotyping Master Mix, 0.125 µL TaqMan probe mix, and 10 ng genomic DNA. The real-time PCR included an initial denaturation step at 95 °C for 10 min, followed by 40 cycles at of 95 °C for 15 s and then at 60 °C for 1 min. For each assay, appropriate controls (nontemplate and known genotype) were included in each typing run to monitor reagent contamination and as a quality control. To validate results from real-time PCR, around 5% of assays were repeated and several cases of each genotype were confirmed by the DNA sequence analysis.

### Statistical analysis

Differences between the 2 groups were considered significant if *p* values were < 0.05. The Mann-Whitney *U*-test and Fisher’s exact test were used to compare differences in distributions of patient demographic characteristics between the cancer-free (control) and UCC groups. The adjusted odds ratios (AORs) and 95% confidence intervals (CIs) of the association between genotype frequencies and risk plus clinicopathological characteristics were estimated using multiple logistic regression models, after controlling for other covariates. We analyzed all data with Statistical Analytic System (SAS Institute, Cary, NC, USA) software (vers. 9.1, 2005) for Windows.

## Results

The distributions of demographic characteristics showed no significant differences in age, gender and tobacco consumption between UCC patients and controls (Table 1). The study population was Taiwanese with male predominant (62.9%) and a low proportion of smokers (25.3% in control and 28.5% in UCC group). In our recruited control group, the frequencies of *CA9* rs2071676 (χ^2^ value: 1.16), rs3829078 (χ^2^ value: 0.31), rs1048638 (χ^2^ value: 0.09), and 376del393 (χ^2^ value: 1.75) were in Hardy-Weinberg equilibrium, respectively. Distribution of CA9 genotypes in Table 2 revealed the most frequent alleles were heterozygous A/G for the +201 (rs2071676) locus and homozygous for A/A, C/C, and Ins/Ins, respectively, for the +1081 (rs3829078), +1584 (rs1048638), and +376 (376del393) loci. We estimated the adjusted odds ratio (AORs) with 95% confidence interval (CIs) by multiple logistic regression models with adjustment for age, gender and tobacco consumption to diminish the possible interferences.

**Table 1:** The distributions of demographical characteristics in 221 controls and 221 patients with urothelial cell carcinoma.

Variable	Controls (N = 221)	Patients (N = 221)	p value
**Age (yrs)**	**Mean ± S.D.**	**Mean ± S.D.**	
	67.85±8.52	68.52±11.79	P = 0.488
**Gender**	**n (%)**	**n (%)**	
Male	139 (62.9%)	139 (62.9%)	
Female	82 (37.1%)	82 (37.1%)	p = 1.000
**Tobacco consumption**			
No	165 (74.7%)	158 (71.5%)	
Yes	56 (25.3%)	63 (28.5%)	p = 0.453
**Stage**			
Superficial tumor (pTa–pT1)		138 (62.4%)	
Invasive tumor (pT2–pT4)		83 (37.6%)	
**Tumor T status**			
T0		64 (29.0%)	
T1-T4		157 (71.0%)	
**Lymph node status**			
N0		204 (92.3%)	
N1+N2		17 (7.7%)	
**Metastasis**			
M0		218 (98.6%)	
M1		3 (1.4%)	
**Histopathologic grading**			
Low grade		34 (15.4%)	
High grade		187 (84.6%)	

’s exact test was used between healthy controls and patients with urothelial cell carcinoma. Mann-Whitney U test or Fisher

**Table 2:** Distribution frequency of *CA9* genotypes in 221 controls and 221 patients with urothelial cell carcinoma.

Variable	Controls (N = 221) n (%)	Patients (N = 221) n (%)	OR (95% CI)	AOR (95% CI)
**rs2071676**				
AA	59 (26.7%)	60 (27.1%)	1.00	1.00
AG	104 (47.1%)	102 (46.2%)	0.964 (0.614–1.515)	0.990 (0.589–1.665)
GG	58 (26.2%)	59 (26.7%)	1.000 (0.600–1.666)	0.987 (0.539–1.809)
AG+GG	162 (73.3%)	161 (72.9%)	0.977 (0.642–1.488)	0.989 (0.608–1.609)
**rs3829078**				
AA	205 (92.8%)	204 (92.3%)	1.00	1.00
AG	16 (7.2%)	17 (7.7%)	1.068 (0.525–2.171)	0.793 (0.344–1.828)
GG	0 (0%)	0 (0%)	--	--
AG+GG	16 (7.2%)	17 (7.7%)	1.068 (0.525–2.171)	0.793 (0.344–1.828)
**rs1048638**				
CC	196 (88.7%)	180 (81.4%)	1.00	1.00
CA	24 (10.9%)	38 (17.2%)	1.724 (0.995–2.987)	2.217 (1.169–4.207)*
AA	1 (0.4%)	3 (1.4%)	3.267 (0.337–31.689)	4.716 (0.363–61.319)
CA+AA	25 (11.3%)	41 (18.6%)	1.786 (1.044–3.055)*	2.303 (1.227–4.320)*
**376del393**				
INS/INS	170 (76.9%)	165 (74.7%)	1.00	1.00
INS/Del	50 (22.6%)	50 (22.6%)	1.030 (0.659–1.611)	0.921 (0.551–1.537)
Del/Del	1 (0.5%)	6 (2.7%)	6.182 (0.736–51.905)	5.532 (0.558–54.844)
INS/Del+ Del/Del	51 (23.1%)	56 (25.3%)	1.131 (0.732–1.749)	1.062 (0.644–1.751)

% confidence intervals (CIs) were estimated by logistic regression models. The adjusted odds ratios (AORs) with their 95% confidence intervals (CIs) were estimated by multiple logistic regression models after controlling for age and gender. * p value < 0.05 as statistically significant. The odds ratios (ORs) and with their 95

Study subjects carrying the C/A genotype of the rs1048638 polymorphism had a significantly increased UCC risk of 2.217 (95% CI = 1.169–4.207, Table 2). Compared to C/C genotype of the rs1048638, C/A+A/A genotype also demonstrated higher UCC risk of 2.303 (95% CI =  = 1.227–4.320). There were no significant increased UCC risk could be found in the polymorphisms of the other three loci rs2071676, rs3829078 and 376del393. Considering cancer stage, UCC patients with the polymorphism of rs1048638 C/A+AA genotype had significantly increased invasive cancer risk of 2.259 (95% CI = 1.136–4.490, Table 3). The data also showed the trend of C/A+A/A genotype patients to have more lymph node or distant metastasis risk. However, no statistical significance could be demonstrated. Among the UCC patients with C/A+A/A genotype of the rs1048638, compared to C/C genotype, smoking increased the invasive cancer risk of 4.750 (95% CI = 1.204–18.746, Table 4).

**Table 3:** Distribution frequency of the clinical status and *CA9* rs1048638 genotype frequencies in 221 patients with urothelial cell carcinoma.

	Genotypic frequencies			
Variable	CC (*N* = 180 ) *n* (%)	CA+ AA (*N* = 41) *n* (%)	OR (95% CI)	p value
**Stage**				
Superficial tumor (pTa–pT1)	119 (66.1%)	19 (46.3%)	1.00	
Invasive tumor (pT2–pT4)	61 (33.9%)	22 (53.7%)	**2.259 (1.136**–4.490)*	p = 0.018*
**Tumor T status**				
T0	53 (29.4%)	11 (26.8%)	1.00	
T1–T4	127 (70.6%)	30 (73.2%)	1.138 (0.531–2.438)	p = 0.739
**Lymph node status**				
N0	169 (93.9%)	35 (85.4%)	1.00	
N1+N2	11 (6.1%)	6 (14.6%)	2.634 (0.913–7.596)	p = 0.065
**Metastasis**				
M0	179 (99.4%)	39 (95.1%)	1.00	
M1	1 (0.6%)	2 (4.9%)	2.463 (0.497–12.219)	p = 0.065
**Histopathologic grading**				
Low grade	27 (15.0%)	7 (17.1%)	1.00	
High grade	153 (85.0%)	34 (82.9%)	0.857 (0.345–2.131)	p = 0.740

**Table 4:** Distribution frequency of the clinical status and *CA9* rs1048638 genotype frequencies in 221 patients with urothelial cell carcinoma with non-smoker and smoker.

	Non-Smoking (n = 158)				Smoking (n = 63)			
Variable	CC (*N* = 128 ) *n* (%)	CA+AA (*N* = 30) *n* (%)	OR (95% CI)	p value	CC (*N* = 52 ) *n* (%)	CA+AA (*N* = 11) *n* (%)	OR (95% CI)	p value
**Stage**								
Superficial tumor (pTa–pT1)	81 (63.3%)	15 (50.0%)	1.00		38 (73.1%)	4 (36.4%)	1.00	
Invasive tumor (pT2–pT4)	47 (36.7%)	15 (50.0%)	1.723 (0.774–3.839)	p = 0.180	14 (26.9%)	7 (63.6%)	**4.750 (1.204**–18.746)*	p = 0.023*
**Tumor T status**								
T0	33 (25.8%)	8 (26.7%)	1.00		20 (38.5%)	3 (27.3%)	1.00	
T1–T4	95 (74.2%)	22 (73.3%)	0.955 (0.388–2.352)	p = 0.921	32 (61.5%)	8 (72.7%)	1.667 (0.395–7.033)	p = 0.476
**Lymph node status**								
N0	120 (93.8%)	26 (86.7%)	1.00		49 (94.2%)	9 (81.8%)	1.00	
N1+N2	8 (6.3%)	4 (13.3%)	2.308 (0.646–8.241)	p = 0.187	3 (5.8%)	2 (18.2%)	2.634 (0.913–7.596)	p = 0.166
**Metastasis**								
M0	128 (99.4%)	29 (96.7%)	1.00		51 (98.1%)	10 (90.9%)	1.00	
M1	0 (0%)	1 (3.3%)	-----	p = 0.067	1 (1.9%)	1 (9.1%)	5.100 (0.294–88.476)	p = 0.283
**Histopathologic grading**								
Low grade	16 (12.5%)	5 (16.7%)	1.00		11 (21.2%)	2 (18.2%)	1.00	
High grade	112 (87.5%)	25 (83.3%)	0.714 (0.239–2.133)	p = 0.545	41 (78.8%)	9 (81.8%)	1.207 (0.227–6.416)	p = 0.823

## Discussion

In the current study, we try to find the association between *CA9* gene polymorphisms and clinicopathological characteristics of UCC. The results showed that patients carrying the C/A genotype of the rs1048638 (3’-UTR regions of the CA9 gene) polymorphism had a significantly increased UCC risk of 2.217 (95% CI = 1.169–4.207). Compared to C/C genotype of the rs1048638, C/A+A/A genotype demonstrated higher UCC risk of 2.303 (95%CI = 1.227–4.320). To consider the tumor invasiveness, patients with C/A+A/A genotype of the rs1048638 polymorphism demonstrated a significant higher invasive caner (pT2-T4) risk of 2.259 (95% CI = 1.136–4.490).

CA9 is abundant in cancers but not in most benign tissues. It helps tumor cells to survive and proliferate in a hypoxic condition [13,21]. The expression of CA9 gene was markedly induced under hypoxic conditions in tumor cells and CA 9 may maintain extracellular acidic pH in microenvironment and help cancer cells grow and metastasize [20]. It was reported that CA9 as a marker of hypoxia and it is also associated with the tumor grade, metastasis and patients prognosis [15,16]. CA9 expression is associated with poor prognosis in many human tumors, and may contribute to aggressive cancer phenotype [17]. In non-small cell lung cancer, the CA9 up-regulation occurs in highly hypoxic/necrotic regions of the tumors and CA9 expression is strongly associated with poor outcome through the mechanism of angiogenesis, apoptosis inhibition, and cell-cell adhesion disruption [19]. In high risk, early stage cervical cancer, CA9 is an independent prognostic factor for both progression free survival and overall survival [26]. Furthermore, it was reported that high expression of CA9 was more frequent with advanced stage in several kinds of solid tumors including bladder cancer [16,27,28]. In the present study, a significantly higher distribution frequency of Invasive tumor stage was exhibited in UCC patients with at least 1 polymorphic A allele of CA9 rs1048638 compared to those with the WT genotype (Table 3). The rs1048638 polymorphism is known in the noncoding 3’-UTR, and as such may affect gene expression through microRNA-targeting sequences. However, the expression and function of CA9 which are affected by this SNP are still unconfirmed. Detailed relevant mechanisms may warrant further studies.

CA9 reflects significant changes in tumor biology and decreased CA9 concentration is independently associated with poor survival in advanced renal cell carcinoma. CA9 can be used to predict clinical outcome in such high risk kidney cancer patients who need adjuvant immunotherapy and CA9 targeted therapies [27]. CA9 also links to poor vascularization and resistance of chemoradiotherapy in head and neck cancer [28] and is associated with reduced overall survival and disease-free survival [29]. Alexander et al [30] review researches of CA9 in renal cell carcinoma (RCC) and conclude it can improve diagnostic accuracy and is an attractive target image of and therapy for clear cell RCC. CA9 expression is suggested to be a strong predictor of recurrence, progression, and overall survival of bladder cancer patients because it was expressed differentially in noninvasive versus invasive tumors, in low-grade versus high-grade bladder cancer, and in primary tumors versus metastases [16].

Alternation of genome can change the cellular phenotype to evolve from a preneoplastic stage into cancer [31]. It is clear that genetics play an important role in carcinogenesis. SNP may influence gene expression, messenger RNA stability and subcellular localization of mRNAs and/or proteins to produce disease [32]. CA9 SNP patients with metastatic clear cell renal cell carcinoma may be associated with better survival and a greater opportunity to response to interleukin-2 [21]. It is also reported that CA9 gene polymorphisms, smoking and betel-quid chewing might alter oral cancer susceptibility and metastasis [13]. Increasing evidences suggest gene polymorphism is associated with the risk and progression of UCC [10,33–40] and can be considered as a prognostic factor of chemoradiotherapy [41]. Cigarette smoking is the most important risk factor for bladder cancer [42]. The present study showed patients with the rs1048638 (3’-UTR regions of the CA9 gene) polymorphism had increased risk of UCC and invasive cancer stage (Table 2 and Table 3). Smoking can increase the risk of invasive cancer in the CA9 SNP UCC patients (Table 4). These lines of evidence suggest that genetic factor and environmental carcinogen exposure were involved in the formation or pathogenesis of UCC.

The study still has some limitations. Firstly, the sample size was small; the statistical significance should be interpreted carefully. Secondly, patients cannot provide detail history of Chinese herbs usage or aromatic amines exposure that might cause bias. We observed the CA9 SNPs affected the clinical behavior of UCC but how this SNP influenced the CAIX expression was still unknown.

In conclusion, this is the first report to study the association between CA9 SNP and UCC risk. The patients carrying the C/A genotype of the rs1048638 of the CA9 gene polymorphism had a significantly increased UCC risk and invasive cancer risk. The synergistic effect of smoking and CA9 gene polymorphisms on the risk of invasive UCC is also demonstrated. Large-scale studies may be required to verify the association between the SNP of hypoxia related gene such as CA9 and UCC risk.
